# Antitumour antibodies induced by rat embryo cells and spontaneous mammary carcinoma cells treated with 3-methylcholanthrene.

**DOI:** 10.1038/bjc.1981.119

**Published:** 1981-06

**Authors:** J. G. Reeve, M. J. Embleton, R. W. Baldwin

## Abstract

It has previously been shown that rat embryo cells treated in vitro with 3-methylcholanthrene (MCA) elicit antibodies in syngeneic rats which react specifically against established MCA-induced sarcomas. To examine the possibility that clonal amplification of one or a few antigenic, preneoplastic clones is responsible for the previously observed specific antibody responses, MCA-treated rat embryo cells have been subjected to 150 Gy of gamma-irradiation before injection into host animals. The resulting antisera were screened for reactivity against a panel of established syngeneic tumours by membrane immunofluorescence and an isotopic antiglobulin test. A positive reaction was observed between an antiserum pool raised against gamma-irradiated MCA-treated cells and the cells of an immunogenic spontaneous mammary carcinoma. Antiserum to gamma-irradiated control (acetone-treated) cells was negative. Thus gamma-irradiation of carcinogen-treated cells before injection failed to abolish specific antibody responses in immunized rats. To investigate further the relationships between cell-carcinogen interaction, neoantigen induction and malignancy, the cells of a non-immunogenic, spontaneous mammary carcinoma were treated with MCA in vitro, and antisera against treated and untreated cells were tested against a panel of established tumours. A positive membrane-immunofluorescence reaction was obtained with an antiserum to MCA-treated cells, but not to untreated cells against an aminoazodye-induced hepatoma, indicating that the previously non-immunogenic mammary carcinoma cells had acquired new antigenic specificities as a consequence of carcinogen treatment.


*Br. J. Cancer* (1981) **43**, 809

### ANTITUMOUR ANTIBODIES INDUCED BY RAT EMBRYO CELLS AND SPONTANEOUS MAMMARY CARCINOMA CELLS TREATED WITH 3-METHYLCHOLANTHRENE

J. G. REEVE, M. J. EMBLETON AND R. W. BALDWIN

*From the Cancer Research Campaign Laboratories, University of Nottingham, Nottingham NG7 2RD*

Received 13 November 1980 Accepted 16 February 1981

**Summary.—It has previously been shown that rat embryo cells treated *in vitro* with 3-methylcholanthrene (MCA) elicit antibodies in syngeneic rats which react specifically against established MCA-induced sarcomas. To examine the possibility that clonal amplification of one or a few antigenic, preneoplastic clones is responsible for the previously observed specific antibody responses, MCA-treated rat embryo cells have been subjected to 150 Gy of γ-irradiation before injection into host animals. The resulting antisera were screened for reactivity against a panel of established syngeneic tumours by membrane immunofluorescence and an isotopic antiglobulin test. A positive reaction was observed between an antiserum pool raised against γ-irradiated MCA-treated cells and the cells of an immunogenic spontaneous mammary carcinoma. Antiserum to γ-irradiated control (acetone-treated) cells was negative. Thus γ-irradiation of carcinogen-treated cells before injection failed to abolish specific antibody responses in immunized rats. To investigate further the relationships between cell–carcinogen interaction, neoantigen induction and malignancy, the cells of a non-immunogenic, spontaneous mammary carcinoma were treated with MCA *in vitro*, and antisera against treated and untreated cells were tested against a panel of established tumours. A positive membrane-immunofluorescence reaction was obtained with an antiserum to MCA-treated cells, but not to untreated cells against an aminoazodye-induced hepatoma, indicating that the previously non-immunogenic mammary carcinoma cells had acquired new antigenic specificities as a consequence of carcinogen treatment.**

Neoplastic transformation by chemical carcinogens often leads to the expression in the transformed cell of neoantigens which are not detectable on normal cells. Classically, these antigens have been defined in tumours by experiments in which syngeneic hosts are pre-immunized with tumour prevented from progressive growth by surgical extirpation or attenuation, followed by challenge with viable tumour cells sufficient to cause progressive growth in controls. Antigens detected by a rejection response in such assays are termed tumour-associated rejection antigens (TARA) or tumour-associated transplantation antigens (TATA). Cell-surface-associated antigens are also detectable on many chemically induced tumours by serological assays involving sera from immune donors (Baldwin *et al.*, 1979) and in general these have the same distribution and selectivity as TARAs. In spite of this concordance of expression, it cannot with certainty be stated that serologically detected antigens are identical with TARAs, and they are therefore better designated separately as tumour-cell surface antigens (TCSA). However, since TARA and TCSA exist in parallel, the general features of their expression are

Correspondence to: Dr M. J. Embleton.

common. Collectively, tumour neoantigens arise as a consequence of the interaction between cell and carcinogen (Embleton & Heidelberger, 1972, 1975) and appear to be closely associated with tumorigenicity (Mondal *et al.*, 1971). However, despite this close association, their acquisition appears not to be an essential step in neoplastic transformation, since many chemically induced tumours and most spontaneous animal tumours have no neoantigens detectable by transplantation tests (Baldwin *et al.*, 1979; Baldwin & Embleton, 1969; Hewitt *et al.*, 1976). On the basis of these observations, the relationship between cell–carcinogen interaction, neoantigen induction and neoplastic transformation are unclear. It has recently been reported that rat embryo cells treated *in vitro* with 3-methylcholanthrene (MCA) acquire new cell-surface antigenic specificities which cross-react specifically and reproducibly with counterparts on certain established chemically induced tumours (Embleton & Baldwin, 1979). These findings suggest that TCSAs of chemically induced tumours are not unique, but may be reproduced on carcinogen-treated cells or their progeny after only a short period of exposure to carcinogen. MCA-treated rat embryo cells failed to undergo neoplastic transformation during the course of these studies, as evidenced by the absence of macroscopic tumours in immunized animals, and extranuclear membrane preparations of MCA-treated cells did not induce antibody responses, indicating that new surface antigens were probably not produced during the short exposure to carcinogen. Thus it seems most likely that neoantigen specificities are acquired during some stage of preneoplastic development occurring after injection of MCA-treated cells into the host. In the present study we have investigated the possibility that clonal amplification of one, or a few, antigenic clones after injection of MCA-treated cells into the host animal is responsible for the observed specific antibody responses. Carcinogen-treated rat embryo cells were therefore subjected to $\gamma$-irradiation before immunization and the resulting antisera were screened against a panel of established rat tumours.

In order to investigate further the relationship between cell–carcinogen interaction, neoantigen expression and malignant transformation we have also treated the cells of non-immunogenic spontaneous mammary carcinoma *in vitro* with MCA, and have looked for specific antibody responses in syngeneic rats immunized with these cells, to determine whether carcinogen treatment would induce neoantigen expression in cells whichare already neoplastic.

### MATERIALS AND METHODS

*Rats.*—Inbred WAB/Not rats were used both for immunological studies and as donors of embryos and syngeneic tumours.

*Carcinogen treatment.*—Single-cell suspensions were prepared from minced 17–19-day rat embryos and from mammary carcinoma Sp15 by repeated treatment with 0·25% trypsin (Difco). Mammary carcinoma Sp15 was of spontaneous origin and is non-immunogenic in syngeneic rats. Between $5 \times 10^7$ and $10^8$ viable embryo or tumour cells were plated in 100mm non-tissue-culture Petri dishes (Oxoid) in 10 ml Eagle's MEM supplemented with calf serum (10%), penicillin (200 i.u./ml), streptomycin (100 $\mu$g/ml) and either 0·5% acetone or 0·5% acetone containing 2 mg/ml 3-methylcholanthrene (MCA, Sigma). The final concentration of MCA in the medium was 10 $\mu$g/ml. Dishes were incubated for 18 h at 37°C in an atmosphere of 5% $CO_2$ in air and the cells harvested by aspiration and rinsing. Cells were subsequently washed $\times 6$ in Hanks' balanced salt solution (HBSS) and recounted.

*$\gamma$-irradiation and immunization.*—Before injection into host animals, rat embryo and Sp15 tumour cells were exposed to 150 Gy $\gamma$-irradiation from a cobalt-60 source. The cells were then injected i.p. into syngeneic male rats, each rat receiving the cellular contents of one dish ($2–5 \times 10^7$ viable cells). Four injections were given at 10-day intervals and the rats bled by cardiac puncture 7 days after the 4th injection. Serum was prepared from clotted blood, and samples from rats receiving identical treatments were pooled and stored at $-20°C$ in small aliquots.

In another experimental design, mammary carcinoma Sp15 cells were treated *in vitro* with either MCA or acetone as previously described, cells were washed $\times 6$ and 1 ml of a suspension containing either $10^6$ viable MCA- or acetone-treated Sp15 cells was injected i.p. into syngeneic animals; all rats developed tumours within 3 weeks. A single-cell suspension of each tumour was then prepared by trypsinization, and 1 ml containing $10^6$ cells of this was injected i.p. into syngeneic rats. Each tumour cell line was subsequently maintained *in vitro* by successive i.p. transplantation of $10^6$ tumour cells. In this way 6 tumour lines were prepared, 3 derived from Sp15 cells treated *in vitro* with MCA and 3 from Sp15 cells treated *in vitro* with acetone.

To investigate whether tumour lines derived from Sp15 cells treated *in vitro* with MCA had acquired immunogenic potential (new antigenic specificities), an antiserum was raised against each tumour line in the following way: at each *in vivo* passage, after removal of $10^6$ cells for line maintenance, the remainder of the tumour single cell suspension was exposed to 150 Gy of $\gamma$-irradiation and $1\text{–}5 \times 10^7$ cells were injected i.p. into each of 7 syngeneic rats. This procedure was repeated $\times 4$, animals being bled by cardiac puncture 7 days after administration of the final inoculum. Serum was prepared and stored as previously described.

*Membrane immunofluorescence test.*—Sera were tested for reactivity against a range of WAB/Not rat tumours using a membrane immunofluorescence test performed on viable suspended cells (Baldwin *et al.*, 1971). Target tumours included an immunogenic spontaneous mammary carcinoma (Sp4), 3 aminoazodye-induced hepatomas (D23, D30 and D192A) and 3 MCA-induced sarcomas (Mc7, Mc106B and Mc107B). Normal rat serum was used as background control and reactivity to MCA-treated cells was expressed as a fluorescence index (FI) defined as:

$$\frac{\begin{matrix}\%\ \text{target cells} \\ \text{unstained by} \\ \text{normal rat serum}\end{matrix} \quad - \quad \begin{matrix}\%\ \text{target cells} \\ \text{unstained by} \\ \text{test serum}\end{matrix}}{\%\ \text{target cells unstained by normal rat serum}}$$

A $\text{FI} \geq 0{\cdot}30$ was taken to indicate a positive reaction (Baldwin *et al.*, 1971).

The specificity of any positive reactions was examined by a series of absorption tests. Aliquots of serum were absorbed for 2 h at 4°C with tumour cells at a density of $10^8$ cells/ml of serum. Cells were removed by centrifugation and the absorbed sera were tested for reactivity against selected target cells. A reduction of more than 50% in FI with absorbed serum compared with that obtained with unabsorbed serum was taken to represent a significant degree of antibody absorption.

*Isotopic antiglobulin test.*—Sera were also tested for reactivity against certain tumour target cells, using an isotopic antiglobulin test (Williams *et al.*, 1977). Dilutions of each antiserum were prepared in HBSS containing 1% bovine serum albumin (BSA, Sigma). 100 $\mu$l aliquots of diluted antiserum were incubated in triplicate with $10^5$ tumour target cells for 1 h on ice. Cells were washed $\times 3$ in HBSS containing 0·1% BSA and incubated for 1 h on ice with 50 $\mu$l of 1% BSA containing $^{125}$I-labelled sheep $F(ab)_2$ anti-rat IgG. Each cell suspension received $\sim 7$ ng protein, to give between $2 \times 10^5$ and $3 \times 10^5$ ct/min. Cells were washed $\times 6$ in HBSS containing 0·1% BSA, centrifuged and the cell pellets counted in a gamma counter.

The mean ct/min obtained with antisera raised to MCA-treated cells were compared with the mean ct/min with antisera to acetone-treated cells, and with normal rat serum. Data were analysed by a single-classification analysis of variance.

*Metabolism of $^3$H-MCA by Sp15 mammary carcinoma cells.*—The ability of mammary carcinoma Sp15 cells to take up and metabolize MCA was determined as described by Diamond *et al.* (1968). Mammary carcinoma Sp15 cells ($5 \times 10^7$) were plated into 100mm Oxoid dishes in 10 ml medium containing 10 $\mu$g/ml MCA, of which 5 ng/ml was $^3$H-MCA (0·5 $\mu$Ci/ml). Cells were incubated at 37°C for 18 h, centrifuged and the supernatant collected. The cell pellet was washed $\times 3$, solubilized in 3M KOH and diluted in 5 vols of methanol before assaying for radioactivity. The supernatant was extracted in a mixture of 20 vols of chloroform: methanol (2:1 v/v) and 4 vols of water. The aqueous and organic solvent fractions of the extraction mixture were separated and assayed for activity in a $\beta$ counter.

After 18 h of incubation, $5 \times 10^7$ cells had bound, on average, 6·6% $^3$H-MCA (6·6 μg/ $5 \times 10^7$ cells). At zero time, 14% of the $^3$H-MCA was recovered in the aqueous phase, after chloroform/methanol extraction. Within 18 h of adding $^3$H-MCA to the cells, 28% of the label in the medium was recoverable in the aqueous phase of the extraction mixture.

### RESULTS

### *Antisera raised against cells treated* in vitro *with MCA or acetone*

As shown in Tables I and II, none of the sera raised against either irradiated acetone-treated rat embryo cells or acetone-treated mammary carcinoma Sp15 cells produced significant membrane immunofluorescence against the tumour target cells studied, indicating that these cells *per se* did not induce antibody responses in syngeneic rats detectable by the membrane-immunofluorescence test. However, when antisera to γ-irradiated MCA-treated cells were tested, two positive reactions were noted. Antiserum A5104, raised against irradiated MCA-treated rat embryo cells, was consistently positive in each of 3 independent tests against an immunogenic spontaneously arising mammary carcinoma, Sp4, and antiserum A4979, raised against MCA-treated mammary carcinoma Sp15 cells, gave significant reactions against an aminoazodye-induced hepatoma, D192A. In the latter case 5 independently repeated tests were consistently positive. In both cases the mean difference in the percentage of cells stained by normal rat serum or acetone control serum and the antiserum to MCA-treated cells was statistically significant as determined by Student's *t* test. All the negative combinations were repeated × 3, with consistently negative results.

TABLE I.—*Membrane immunofluorescence reactions against rat tumours by antiserum to MCA-treated, irradiated embryo cells*

| | | Mean FI (±s.d.) — Antisera to irradiated embryo cells treated with: | |
|---|---|---|---|
| Target cells† | | MCA (A5104) | Acetone (A5105) |
| Hepatoma | D23 | 0·12 ±0·02 | 0·00* |
| | D30 | 0·02 ±0·01 | 0·02±0·02 |
| | D192A | 0·06 ±0·02 | 0·00* |
| Sarcoma | Mc7 | 0·18 ±0·01 | 0·00* |
| | Mc106B | 0·00*±0·00 | 0·00 |
| | Mc107B | 0·23 ±0·01 | 0·00 |
| Mammary carcinoma | Sp4 | 0·34§±0·14 | 0·09±0·01 |
| | Sp15 | 0·03¶ | 0·13¶ |

† Hepatomas were originally induced by oral 4-dimethylaminoazobenzene. The sarcomas were induced by s.c. injection of 3-methylcholanthrene, and breast carcinoma Sp4 was spontaneous.
* Values numerically lower than zero.
§ $P < 0·025$.
¶ Results from one test only.

TABLE II.—*Membrane immunofluorescence reactions against rat tumours by syngeneic antiserum to γ-irradiated MCA-treated mammary carcinoma Sp15 cells*

| | | Mean FI (±s.d.) — Antisera to Sp15 cells treated with: | |
|---|---|---|---|
| Target cells† | | MCA (A4979) | Acetone (A4980) |
| Hepatoma | D23 | 0·13 ±0·02 | 0·01±0·01 |
| | D30 | 0·21 ±0·18 | 0·05±0·04 |
| | D192A | 0·32‡±0·05 | 0·08±0·06 |
| Sarcoma | Mc7 | 0·10 ±0·01 | 0·10±0·01 |
| | Mc106B | 0·06 ±0·08 | 0·00 |
| | Mc107B | 0·15 ±0·13 | 0·12±0·16 |
| Carcinoma | Sp4 | 0·11 ±0·12 | 0·09±0·12 |
| | Sp15§ | 0·11 | 0·02 |

† See Table I.
‡ $P < 0·0005$ derived from 5 independent tests.
§ Results from one test only.

The relatively high mean FI of 0·21 which was obtained when A4979 was reacted with hepatoma D30 is considered spurious, since a positive reaction was obtained in only 1 of the 3 tests performed.

The specificity of the observed antibody responses was confirmed by the absorption tests shown in Table III. Thus the reactivity of serum A5104 against mammary carcinoma Sp4 could be removed by absorption with Sp4 cells, but not by absorption with either hepatoma D23 or sarcoma Mc7 cells. Similarly the reactivity of serum A4979 against hepatoma

TABLE III.—*Absorption of anti-tumour antibodies from antisera to MCA-treated rat embryo and MCA-treated mammary carcinoma Sp15 cells*

| Target cells | Serum* | Absorbing cells† | FI | % FI reduction |
|---|---|---|---|---|
| Sp4 | Anti-MCA-treated embryo cells (A5104) | None | 0·36 | — |
| | | D23 | 0·30 | 17 |
| | | Mc7 | 0·32 | 12 |
| | | Sp4 | 0·00 | 100 |
| D192A | Anti-MCA-treated Sp15 cells (A4979) | None | 0·41 | — |
| | | Mc106B | 0·35 | 15 |
| | | D192A | 0·06 | 85 |

* Antisera raised against γ-irradiated cells.
† Serum was absorbed for 2 h at 4°C using $10^8$ cells/ml of serum, which were removed by centrifugation at 10,000 *g* for 10 min.

D192A was removed by absorption with D192A cells but not by absorption with the cells of sarcoma Mc106B.

The positive membrane-immunofluorescence reaction between A5104 and Sp4 and the specificity of this reaction have been confirmed using the isotopic antiglobulin test. Thus in 3 independent tests the mean ct/min when A5104 was reacted with Sp4 target cells was significantly higher than that obtained with either the acetone control antiserum (A5105) or normal rat serum (Table IV). Both antisera A5104 and A5105 failed to react significantly with 5 other syngeneic tumours tested (D23, D192A, Mc7, Mc106B and Mc107B). Table V shows that the reactivity of antiserum A5104 against Sp4 cells was removed by absorption with Sp4 cells, as indicated by a significant

TABLE V.—*Absorption of Sp4 reactivity from syngeneic antisera to irradiated MCA-treated rat embryo cells*

| Antiserum raised against | Absorption | Binding of $^{125}$I sheep F(ab)$_2$ anti-rat IgG (Mean ct/min ± s.e.) |
|---|---|---|
| Irradiated MCA-treated embryo cells | — | 1006 ± 74 |
| | Sp4 | 709 ± 26 |
| | D23 | 1005 ± 38 |
| Normal rat serum | — | 475 ± 31 |

reduction in the mean ct/min, but not by absorption with the cells of hepatoma D23, thus confirming the findings from membrane immunofluorescence.

*Antisera raised against tumour lines derived from Sp15 cells treated* in vitro *with MCA or acetone*

When antisera to Sp15 tumour lines derived from cells treated *in vitro* with acetone were tested against a panel of established chemically induced and spontaneous tumours (including mammary carcinoma Sp15), no positive membrane-immunofluorescence reactions were noted (Table VI). Furthermore, antisera raised to γ-irradiated, untreated Sp15 cells also failed to give positive membrane-immunofluorescence reactions against either Sp15 or other tumour cells tested in this study. However, a positive membrane-immunofluorescence reaction was observed between one antiserum raised against a tumour line derived from Sp15 cells

TABLE IV.—*Antiserum reactions against mammary carcinoma Sp4 target cells as determined by an isotopic-antiglobulin test**

| Antisera raised against | Dilution | Binding of $^{125}$I sheep F(ab)$_2$ anti-rat IgG (Mean ct/min ± s.e.) Test 1 | Test 2 | Test 3 |
|---|---|---|---|---|
| Irradiated MCA-treated embryo cells (A5104) | 1/2 | 1006 ± 74 | 5322 ± 359 | 2098 ± 186 |
| | 1/5 | 591 ± 61 | 3710 ± 36 | 1455 ± 59 |
| Irradiated acetone-treated cells (A5105) | 1/2 | 615 ± 39 | 2614 ± 22 | 1131 ± 104 |
| | 1/5 | 485 ± 170 | 2673 ± 149 | 1018 ± 44 |
| Normal rat serum | 1/2 | 475 ± 31 | 852 ± 161 | 710 ± 15 |
| | 1/5 | 467 ± 27 | 1114 ± 77 | 861 ± 9 |

* See Materials and Methods.
Data were analysed by single-classification analysis of variance: the mean ct/min with A5104 was significantly higher than that obtained with either A5105 or normal rat serum ($P < 0·01$).

TABLE VI.—*Membrane-immunofluorescence reactions against rat tumours by antiserum to tumours derived from mammary carcinoma Sp15 cells treated* in vitro *with MCA*

| Target cells† | | Mean FI (±s.d.) Antisera to Sp15 tumours derived from cells treated *in vitro* with MCA (A5764) | Acetone (A5763) |
|---|---|---|---|
| Hepatoma | D23 | 0·00 | 0·00 |
| | D192A | 0·00 | 0·00 |
| Sarcoma | Mc7 | 0·01 ± 0·00 | 0·01 ± 0·14 |
| | Mc107B | 0·00 | 0·01 ± 0·01 |
| | Mc109B | 0·19 ± 0·06 | 0·09 ± 0·07 |
| Mammary carcinoma | Sp4 | 0·63 ± 0·05‡ | 0·16 ± 0·05 |
| | Sp15§ | 0·00 | 0·00 |

† See Table I.
‡ $P < 0{\cdot}005$ derived from 4 independent tests.
§ Results from one test only.

treated *in vitro* with MCA, and the cells of an immunogenic spontaneous mammary carcinoma, Sp4 (Table IV). This antiserum, A5764, was consistently positive in each of 3 independent tests, and the specificity of the reactions was confirmed by a series of absorption tests (Table VII). Thus the reactivity of serum A5764 against mammary carcinoma Sp4 was removed by absorption with Sp4 cells, but not by absorption with the cells of hepatoma D23, sarcoma Mc7 or carcinoma Sp15.

TABLE VII.—*Absorption of anti-tumour antibodies from antisera to tumours derived from mammary carcinoma Sp15 cells treated* in vitro *with MCA*

| Target cells | Serum* | Absorbing cells† | FI | % FI reduction |
|---|---|---|---|---|
| Sp4 | Anti-MCA/Sp15 tumour (A5764) | None | 0·71 | — |
| | | Mc7 | 0·52 | 27 |
| | | D23 | 0·54 | 24 |
| | | Sp15 | 0·65 | 8 |
| | | Sp4 | 0·12 | 83 |

* Raised against γ-irradiated cells of tumours derived from Sp15 cells treated *in vitro* with MCA.
† Serum absorbed for 2 h at 4°C using $10^8$ cells/ml, which were removed by centrifugation at 10,000 *g* for 10 min.

The positive reaction between antiserum A5764 and Sp4 was also confirmed by the isotopic antiglobulin test. In 2 independent tests the mean ct/min when A5764 was reacted against Sp4 target cells was significantly higher than that obtained

TABLE VIII.—*Reactivity against mammary carcinoma Sp4 by antiserum to tumour derived from mammary carcinoma Sp15 cells treated* in vitro *with MCA*

| Serum* | Binding of $^{125}$I sheep $F(ab)_2$ anti-rat IgG to serum-treated Sp4 cells Test 1 | Test 2 |
|---|---|---|
| Anti-MCA/Sp15-treated tumour line (A5764) | 4541 ± 575 | 1111 ± 97 |
| Anti-AC/Sp15 (A5763) | 1222 ± 98 | 667 ± 59 |
| Normal rat serum | 1479 ± 135 | 517 ± 42 |

Data were analysed by a single-classification analysis of variance: the mean ct/min when A5764 was reacted against Sp4 target cells is significantly higher than that obtained with either acetone control serum (A5763) or normal rat serum ($P < 0{\cdot}01$).

with either the acetone control serum, A5763, or normal rat serum (Table VIII).

The 4 remaining antisera directed against Sp15 tumour lines derived from cells treated *in vitro* with MCA or acetone failed to give positive reactions, as determined by the membrane-immunofluorescence test and the isotopic-antiglobulin test, against either Sp4 or the chemically induced tumours included in this study.

One explanation for the reactivity between antisera raised to carcinogen-treated cells and certain chemically induced or spontaneous tumours is that it is due to the presence of natural antibody rather than to the induction of neoantigens on carcinogen-treated cells. This possibility was examined by screening both D192A and Sp4 against 10 samples of serum from normal rats. Reactivity of the normal rat serum samples was assayed with both membrane immunofluorescence and the isotopic antiglobulin tests. However, no reactivity was found between the target cells and the samples of normal rat serum by either method.

### DISCUSSION

It has previously been shown that rats immunized with embryo cells treated *in vitro* with MCA develop antibodies which react specifically and reproducibly with certain chemically induced rat tumours (Embleton & Baldwin, 1979). On the basis of this finding it seems highly likely that the TCSAs of chemically induced tumours are not unique, but can be reproduced on carcinogen-treated cells or their progeny after a short period of exposure to carcinogen. In the present study, an antiserum raised to $\gamma$-irradiated MCA-treated rat embryo cells reacted specifically and reproducibly with the cells of an immunogenic spontaneous mammary carcinoma, Sp4. This finding suggests that the potential repertoire of neoantigen specificities which may be expressed on rat embryo cells as a result of carcinogen treatment is not restricted to those antigenic specificities demonstrable as TCSA on chemically induced tumours, but also includes antigens which cross-react with counterparts on the cells of a spontaneous tumour.

It has been postulated that the new antigens induced on carcinogen-treated cells arise during some stage of preneoplastic development (Embleton & Baldwin, 1979). Thus it is possible that the specific antibody responses observed in rats immunized with carcinogen-treated cells develop as one or few preneoplastic antigenic clones undergo amplification after injection into the host animal as a result of further *in vivo* development. However, in the present study, $\gamma$-irradiation of carcinogen-treated cells before injection into host animals failed to abolish specific antibody responses in immunized animals. This finding makes clonal amplification of preneoplastic antigenic clones an unlikely requirement for the generation of the observed specific antibody responses, since after $\gamma$-irradiation cells are incapable of repeated division. An alternative hypothesis is that neoantigens appear through early interactions of carcinogen with target cells by a mechanism not involving multiple cell divisions, and that in carcinogenesis such antigens persist through all stages of progression to malignancy. This view is supported by the observation that skin papillomas induced with MCA express neoantigens with specificities identical to TARAs on subsequent skin carcinomas (Lappé, 1969).

The results obtained when the cells of mammary carcinoma Sp15 were incubated in the presence of $^3$H-MCA indicate that these cells are able to bind and metabolize MCA under the culture conditions previously described. Also an antiserum raised to MCA-treated Sp15 cells reacted consistently and reproducibly with the cells of an aminoazodye-induced hepatoma, D192A. One interpretation of these findings is that as a result of carcinogen treatment these previously non-immunogenic cells have acquired neoantigen specificities which are absent from untreated cells, and if so, that, as previously suggested by Embleton & Baldwin (1979), chemically induced neoantigens are not specific for a particular carcinogen. Furthermore, these results indicate that neoantigen specificities can be induced on already neoplastic cells.

In the second experimental design, an antiserum raised against a tumour derived from Sp15 cells treated *in vitro* with MCA reacted consistently and reproducibly with the cells of the spontaneous immunogenic mammary carcinoma, Sp4. The corresponding tumour derived from acetone-treated Sp15 cells failed to elicit Sp4 antiserum reactivity, suggesting that the observed reactivity of antiserum A5764 with Sp4 cells arose through the *in vitro* treatment of Sp15 cells with MCA. One interpretation of this finding is that the carcinogen-treated Sp15 tumour cells have acquired an antigenic specificity which is the same as, or similar to, the TCSA of Sp4.

Since the experimental design used in this study makes it possible to reclaim carcinogen-treated cells as overt tumour, following injection into host animals, we should now be able to test directly whether or not cells treated *in vitro* with carcinogen do indeed express TCSA, and perhaps whether TCSA and TARA are identical. Previous studies have shown that carcinogen-treated rat embryo cells, while capable of expressing neoantigens which cross-react with their counterparts on certain established tumours, failed to manifest malignancy during the course of these studies (Embleton & Baldwin, 1979). This observation, together with the apparent induction of neoantigens on already neoplastic cells, may indicate that the induction of new antigenic specificities by MCA is not necessarily related to the change from the normal to the neoplastic phenotype.

This work was supported by the Cancer Research Campaign, U.K.

REFERENCES

BALDWIN, R. W., BARKER, C. R., EMBLETON, M. J., GLAVES, D., MOORE, M. & PIMM, M. V. (1971) Demonstration of cell-surface antigens on chemically-induced tumours. *Ann. N.Y. Acad. Sci.*, **177**, 268.

BALDWIN, R. W. & EMBLETON, M. J. (1969) Immunology of spontaneously arising rat mammary adenocarcinomas. *Int. J. Cancer*, **4**, 430.

BALDWIN, R. W., EMBLETON, M. J. & PIMM, M. V. (1979) Neoantigens in chemical carcinogenesis. In *Carcinogens: Identification and Mechanisms of Action*. Ed. Griffin & Shaw. New York: Raven Press. p. 365.

DIAMOND, L., SARDET, C. & ROTHBLAT, G. H. (1968) The metabolism of 7,12-dimethylbenz(a)anthracene in cell cultures. *Int. J. Cancer*, **3**, 838.

EMBLETON, M. J. & BALDWIN, R. W. (1979) Tumour related antigen specificities associated with 3-methylcholanthrene-treated rat embryo cells. *Int. J. Cancer*, **23**, 840.

EMBLETON, M. J. & HEIDELBERGER, C. (1972) Antigenicity of clones of mouse prostate cells transformed *in vitro*. *Int. J. Cancer*, **9**, 8.

EMBLETON, M. J. & HEIDELBERGER, C. (1975) Neoantigens on chemically transformed cloned C3H mouse embryo cells. *Cancer Res.*, **35**, 2049.

HEWITT, H. B., BLAKE, E. R. & WALDER, A. S. (1976) A critique of the evidence for active host defence against cancer, based on personal studies of 27 murine tumours of spontaneous origin. *Br. J. Cancer*, **33**, 241.

LAPPÉ, M. A. (1969) Tumour specific transplantation antigens: Possible origin in premalignant lesions. *Nature*, **223**, 82.

MONDAL, S., EMBLETON, M. J., MARQUARDT, H. & HEIDELBERGER, C. (1971) Production of variants of decreased malignancy and antigenicity from clones transformed *in vitro* by methylcholanthrene. *Int. J. Cancer*, **8**, 410.

WILLIAMS, A. F., GALFRÉ, G. & MILSTEIN, C. (1977) Analysis of cell surfaces by xenogeneic myeloma-hybrid antibodies: Differentiation antigens of rat lymphocytes. *Cell*, **12**, 663.


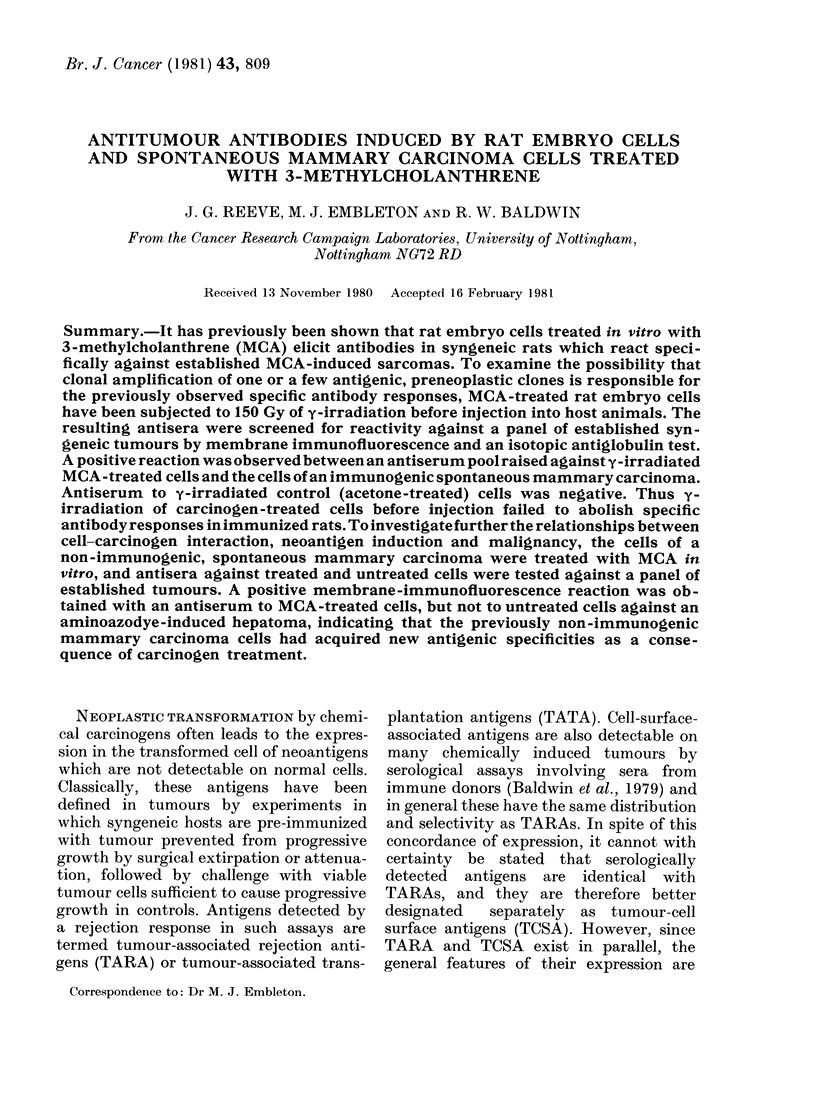

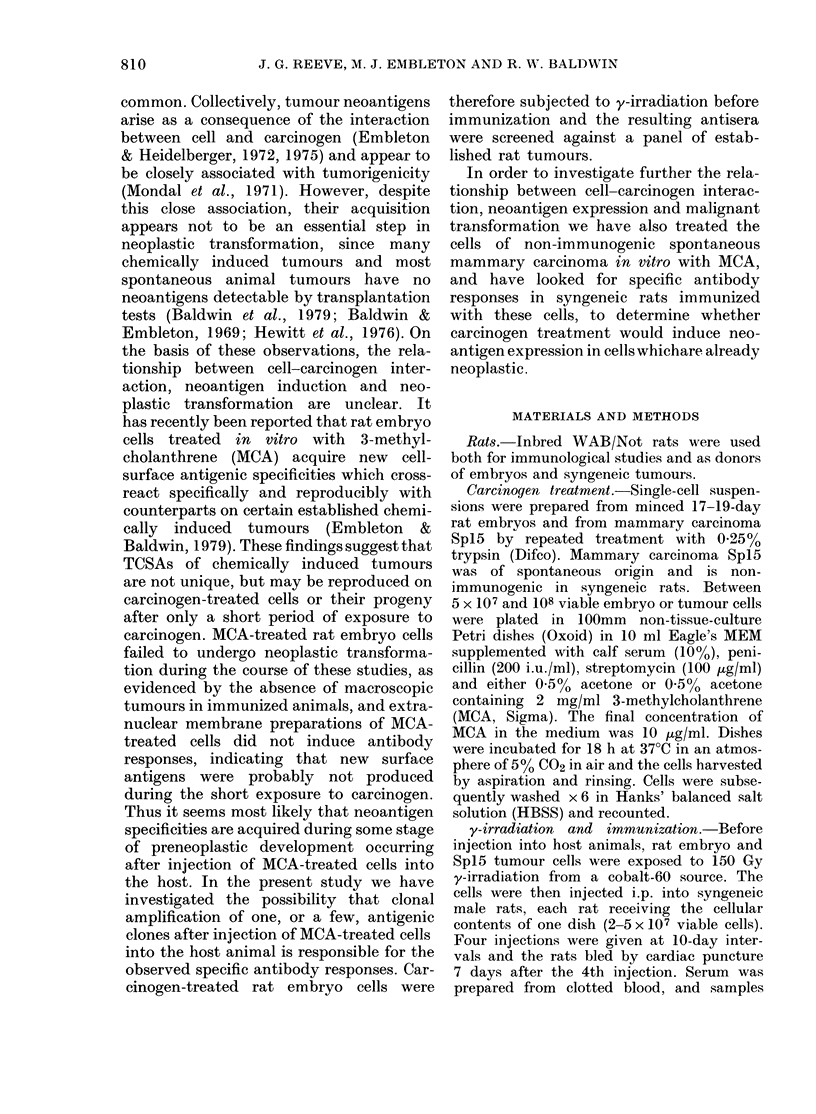

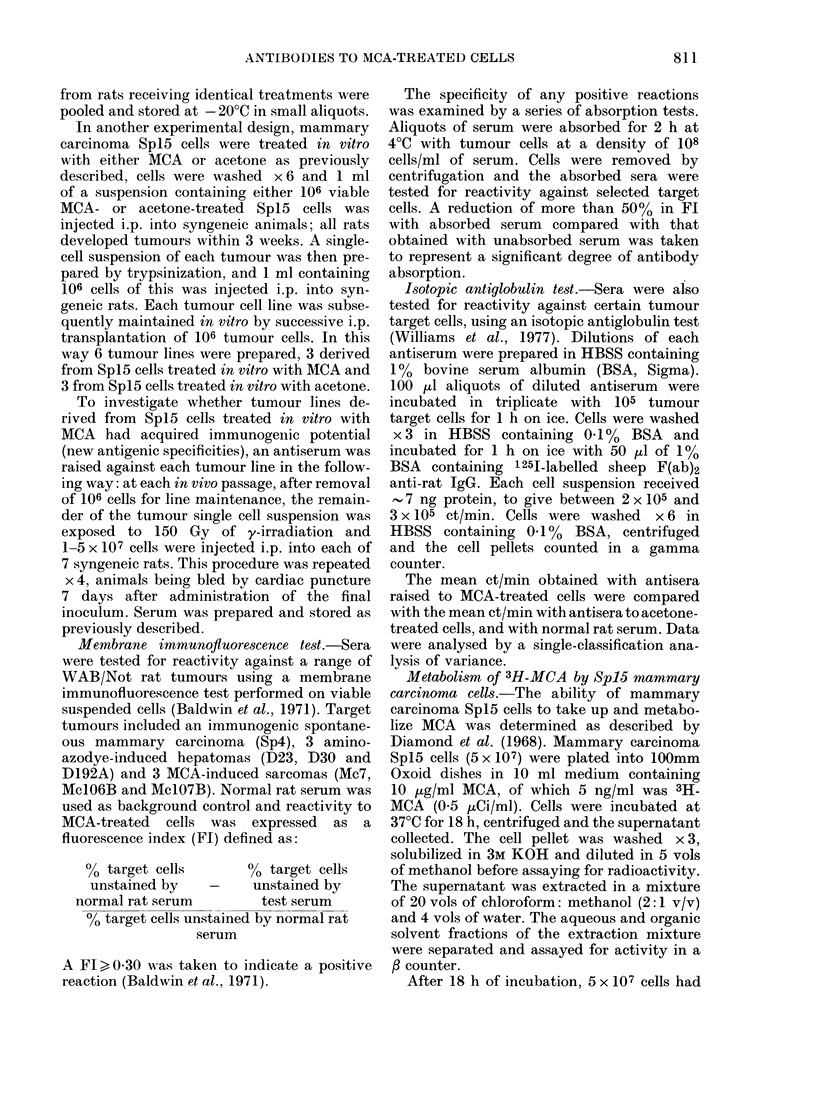

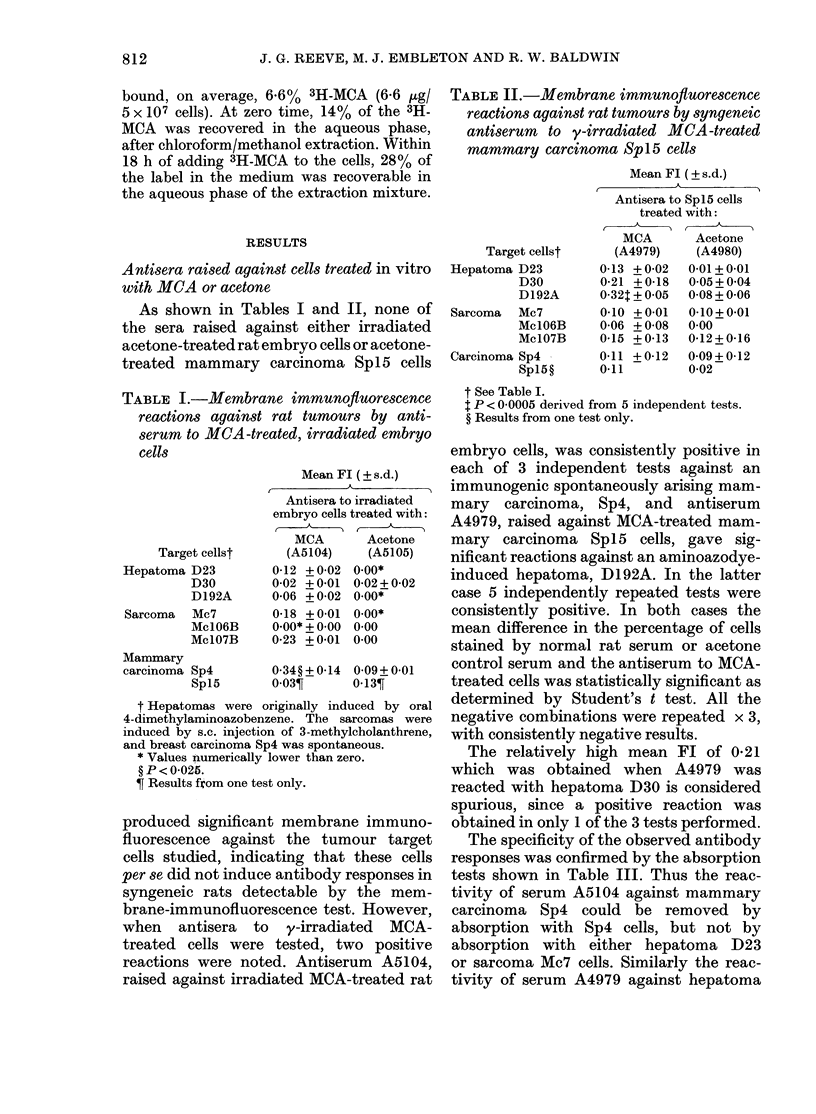

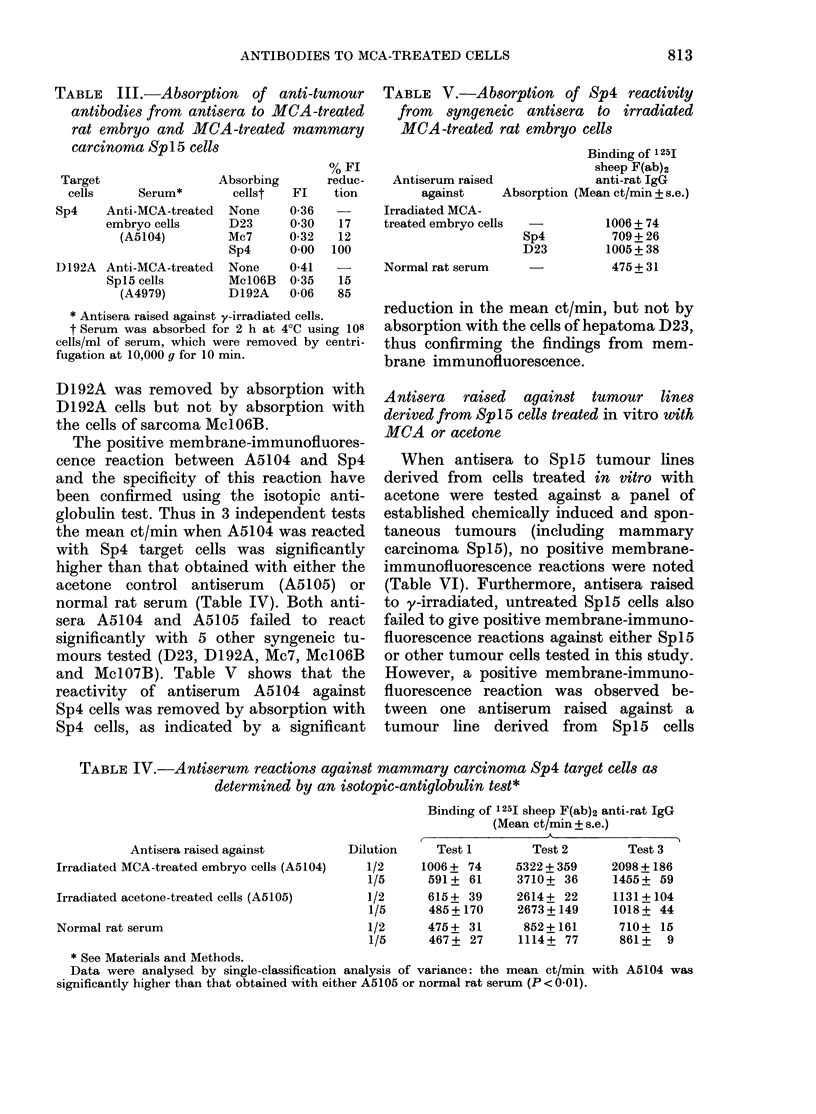

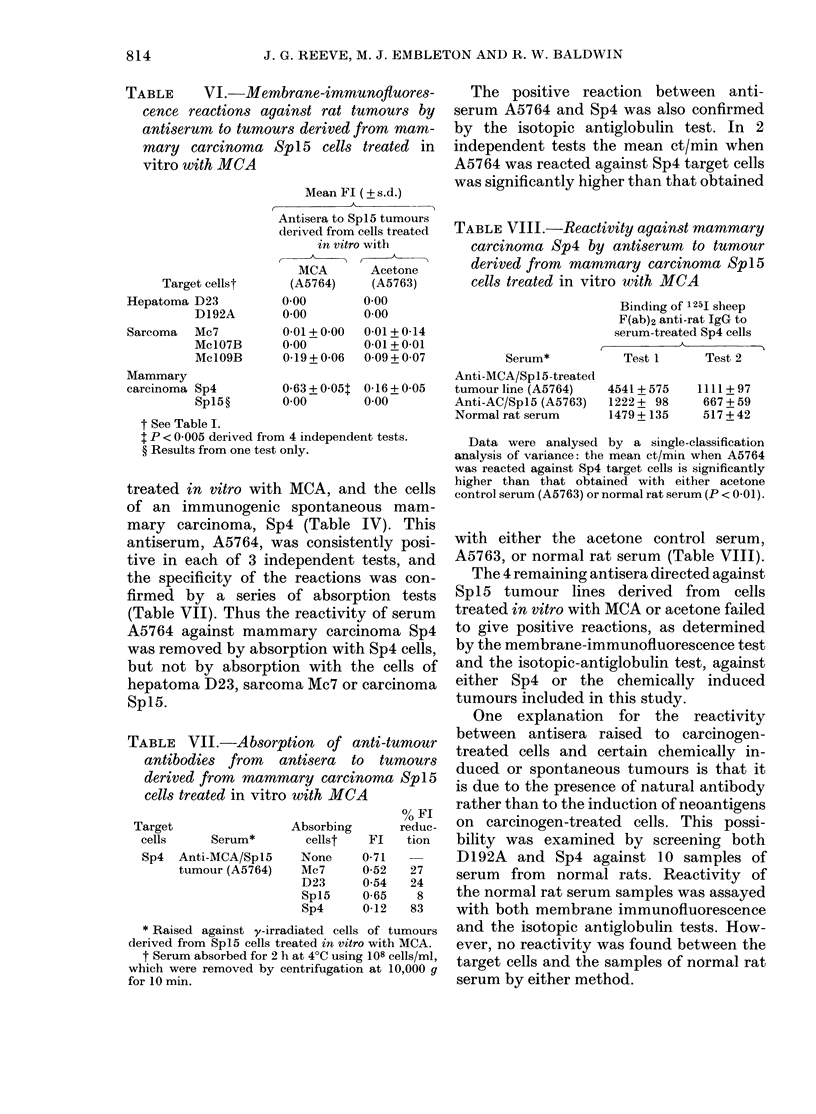

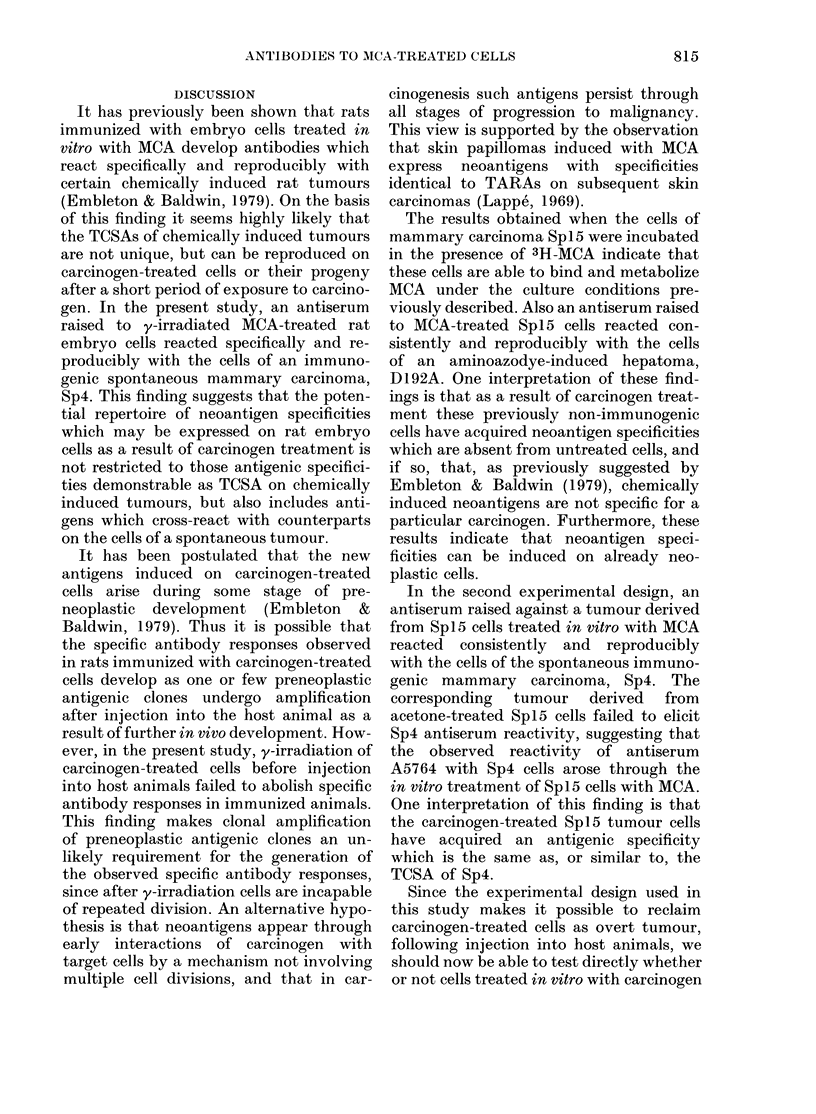

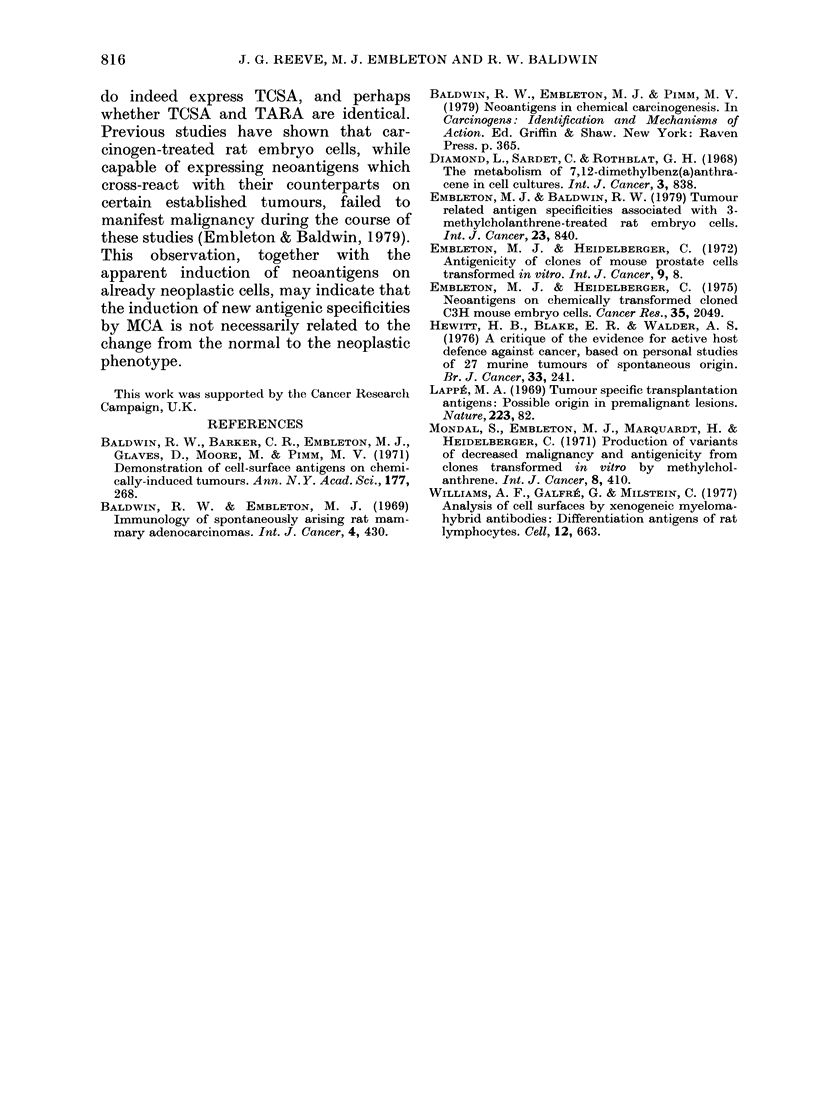


## References

[OCR_01006] Baldwin R. W., Barker C. R., Embleton M. J., Glaves D., Moore M., Pimm M. V. (1971). Demonstration of cell-surface antigens on chemically induced tumors.. Ann N Y Acad Sci.

[OCR_01013] Baldwin R. W., Embleton M. J. (1969). Immunology of spontaneously arising rat mammary adenocarcinomas.. Int J Cancer.

[OCR_01025] Diamond L., Sardet C., Rothblat G. H. (1968). The metabolism of 7,12-dimethylbenz(a)anthracene in cell cultures.. Int J Cancer.

[OCR_01030] Embleton M. J., Baldwin R. W. (1979). Tumour-related antigen specificities associated with 3-methylcholanthrene-treated rat embryo cells.. Int J Cancer.

[OCR_01036] Embleton M. J., Heidelberger C. (1972). Antigenicity of clones of mouse prostate cells transformed in vitro.. Int J Cancer.

[OCR_01041] Embleton M. J., Heidelberger C. (1975). Neoantigens on chemically transformed cloned C3H mouse embryo cells.. Cancer Res.

[OCR_01046] Hewitt H. B., Blake E. R., Walder A. S. (1976). A critique of the evidence for active host defence against cancer, based on personal studies of 27 murine tumours of spontaneous origin.. Br J Cancer.

[OCR_01053] Lappé M. A. (1969). Tumour specific transplantation antigens: possible origin in pre-malignant lesions.. Nature.

[OCR_01058] Mondal S., Embleton M. J., Marquardt H., Heidelberger C. (1971). Production of variants of decreased malignancy and antigenicity from clones transformed in vitro by methylcholanthrene.. Int J Cancer.

[OCR_01065] Williams A. F., Galfrè G., Milstein C. (1977). Analysis of cell surfaces by xenogeneic myeloma-hybrid antibodies: differentiation antigens of rat lymphocytes.. Cell.

